# Mito-Modulatory Medication Use and Skeletal Muscle Bioenergetics Among Older Men and Women: The Study of Muscle, Mobility, and Aging

**DOI:** 10.1093/gerona/glaf063

**Published:** 2025-03-23

**Authors:** Howard J Phang, Jaclyn Bergstrom, Rabia S Atayee, Laura A Hart, Peggy M Cawthon, Terri Blackwell, Philip A Kramer, Giovanna Distefano, Erin E Kershaw, Steven R Cummings, Anthony J A Molina

**Affiliations:** Department of Medicine, University of California San Diego, La Jolla, California, USA; Skaggs School of Pharmacy and Pharmaceutical Sciences, University of California San Diego, La Jolla, California, USA; Department of Medicine, University of California San Diego, La Jolla, California, USA; Skaggs School of Pharmacy and Pharmaceutical Sciences, University of California San Diego, La Jolla, California, USA; Department of Pharmacy, University of Washington School of Pharmacy, Seattle, Washington, USA; Department of Epidemiology and Biostatistics, University of California, San Francisco, San Francisco, California, USA; San Francisco Coordinating Center, California Pacific Medical Center Research Institute, San Francisco, California, USA; San Francisco Coordinating Center, California Pacific Medical Center Research Institute, San Francisco, California, USA; Department of Internal Medicine-Gerontology and Geriatric Medicine, Wake Forest University School of Medicine, Winston-Salem, North Carolina, USA; Translational Research Institute, AdventHealth, Orlando, Florida, USA; Division of Endocrinology and Metabolism, Department of Medicine, University of Pittsburgh, Pittsburgh, Pennsylvania, USA; Department of Epidemiology and Biostatistics, University of California, San Francisco, San Francisco, California, USA; Department of Medicine, University of California San Diego, La Jolla, California, USA; (Medical Sciences Section)

**Keywords:** Medicine, Mitochondria, Polypharmacy

## Abstract

**Background:**

The potential impacts of drug-induced modulation of mitochondrial function in humans remain unclear despite the high prevalence of “mito-modulatory” medication use among older adults. Although these medications, such as statins and metformin, have undergone extensive characterization of their effects on mitochondrial function in vitro, the effects in humans are far more complex and poorly understood.

**Methods:**

This study uses data from the Study of Muscle, Mobility, and Aging (SOMMA) to evaluate how mito-modulatory medication use is related to skeletal muscle bioenergetic capacity, measured by ex vivo high-resolution respirometry and in vivo phosphorus magnetic resonance spectroscopy in healthy older adults.

**Results:**

We found that mito-modulatory medication use was related to lower maximal complex I & II supported oxidative phosphorylation (Max OXPHOS), maximal electron transfer system capacity (Max ETS), and maximal ATP production capacity (ATP Max) in men, but not in women. We also found this to be dependent on the number of medications used, in which higher mito-modulatory medication load was associated with lower Max OXPHOS, Max ETS, and ATP Max.

**Conclusions:**

Our results provide greater insight into the potential clinical effects of mito-modulatory medication use and highlight the need to test the impact of these medications on mitochondrial function in randomized trials.

Drug-induced modulation of mitochondrial function has been described in preclinical models for many different medications, such as hydroxymethylglutaryl-coenzyme A reductase inhibitors (statins) for hypercholesterolemia and metformin for diabetes, among others (1–9). Such effects encompass a variety of mitochondrial changes including altered respiratory capacity, membrane potential, expression and activity of electron transfer system (ETS) complexes, and regulation of oxidative stress. In some cases, these mitochondrial effects are unrelated to the therapeutic action of the medication and are instead discovered as unintended side effects. Despite this, these “mito-modulatory” medications remain routinely prescribed for older adults; for instance, in 2022, metformin and atorvastatin were the top 2 most prescribed medications in the United States, with over 80 million estimated prescriptions each (10). Given the high rate of prescription medication use among older adults, examining the degree of mitochondrial modulation associated with these common medications is critical to understanding their clinical consequences.

Important gaps in knowledge pertaining to the use of mito-modulatory medications remain unaddressed. Although mito-modulatory effects have been characterized in multiple cell types, tissues, and model systems, the findings are largely within a preclinical context (11). Outcomes in human studies are far more complicated; a “toxic” in vitro effect does not always directly translate to a detrimental in vivo effect, and vice versa. For instance, it is well established in vitro that metformin inhibits complex I and decreases oxidative phosphorylation (OXPHOS)-mediated adenosine triphosphate (ATP) production, but it has also been found to support mitochondrial autophagy, biogenesis, and bioenergetic capacity in humans (9,12,13). The impact of mito-modulatory medication exposure on mitochondrial function in humans and the resulting clinical implications remain unclear. Further, the few small studies that have examined mito-modulatory medications in humans tend to focus on individual medications or medication classes, rather than the effect of combined or total mito-modulatory medication exposure. Polypharmacy is an important issue to investigate, as it is common—a cross-sectional study of the U.S. Centers for Disease Control and Prevention’s National Ambulatory Medical Care Survey from 2009 to 2016 found that 65% of older adult patients were on at least 2 prescription medications, and 37% were on 5 or more prescription medications (14). It is also well established that increased medication load is associated with higher rates of adverse events and hospitalizations (15). With regard to mito-modulatory medications, preclinical studies have identified heightened effects of combined mito-modulatory medication exposures, akin to clinical drug–drug interactions (16).

Skeletal muscle is an energy-hungry organ system that relies heavily on mitochondrial energy production, and skeletal muscle mitochondrial dysfunction has been linked to decline in physical performance in older adults (17–19). Multiple lines of research are being undertaken to understand changes in skeletal muscle bioenergetics with age. High-resolution respirometry remains a robust method to measure mitochondrial function ex vivo, providing insight on mitochondrial oxygen utilization with specific contributions of ETS complexes. The respiratory capacity of human skeletal muscle has been correlated with aging and age-related phenotypes (20–22). Another less invasive method, phosphorus magnetic resonance spectroscopy (31P-MRS), provides a different but complementary in vivo measure of OXPHOS capacity through phosphocreatine (PCr) recovery and modeled ATP production (23). Together, ex vivo respirometry of permeabilized vastus lateralis skeletal muscle fibers and in vivo 31P-MRS of quadriceps muscle provide a comprehensive assessment of skeletal muscle mitochondrial bioenergetics and may be valuable tools for understanding whether drug-induced mitochondrial modulation affects human skeletal muscle bioenergetics.

The goal of the present study was to assess how mito-modulatory medication use is related to measures of skeletal muscle bioenergetics in older adults using data from the Study of Muscle, Mobility, and Aging (SOMMA). We hypothesized that mito-modulatory medication use is associated with lower skeletal muscle bioenergetic capacity measured by ex vivo high-resolution respirometry and in vivo 31P-MRS.

## Method

### Study Design

SOMMA is a multicenter, prospective longitudinal cohort study designed to investigate the biological basis of human aging with an emphasis on mobility decline and skeletal muscle. A total of 879 participants were recruited and consented between April 2019 and December 2021. Informed consent was obtained after the nature and possible consequences of the study were explained. At baseline, participants completed questionnaires and exams over 3 clinic visits. The 3 visits were conducted within 6 to 12 weeks from the first visit. Follow-up visits consisting of repeated assessments are ongoing. SOMMA implements strict inclusion criteria including age of 70 or older, body mass index (BMI) less than or equal to 40 kg/m^2^, dementia-free, and others. Importantly, participants were eligible if they were willing and able to undergo muscle tissue biopsy, magnetic resonance (MR) spectroscopy, walk 400 m, and had no advanced disease. Further details of SOMMA can be found in a previous publication (24).

The present study utilized a subset of SOMMA participants (448) with consistent medication use between baseline days and complete bioenergetic and covariate measurements. Consistent medication use refers to whether participants were consistent in their use of the reported mito-modulatory medication (ie, if that medication was brought and verified on zero or all 3 baseline visits). For example, if a participant brought that medication to only 1 or 2 baseline visits, that would be considered inconsistent use. We included participants with medication use information, with consistent medication use between baseline days, with ≤90 days between all baseline days, with Max OXPHOS and Max ETS muscle respirometry, with ATP Max, and with complete covariates as outlined in Supplementary Figure S1. From this sample, we identified individuals using mito-modulatory medications from their medication history.

### Medication History

Medication use history within 30 days prior was collected during 3 baseline clinic visits. Participants were asked to bring prescription medications during each visit, and medication information was recorded in an electronic database, verified by examination of medication containers. Participants who did not bring all medications were contacted by clinical staff via telephone or a return visit to provide medical information. For each medication, the following information was recorded: duration of use, formulation, and frequency of use. Each medication was matched to a medication code and generic ingredient name(s) using the RxTerms Dictionary (National Library of Medicine, Bethesda, MD), a drug interface terminology derived from RxNorms for medication history recording, as well as RxMiX for matching the medications to the WHODrug Anatomical Therapeutic Chemical (ATC) classification system (Uppsala Monitoring Centre, Uppsala, Sweden) (25–28).

### Vastus Lateralis Muscle Biopsy

Biopsies of vastus lateralis muscle were taken to provide fiber bundles for high-resolution respirometry. Briefly, biopsy specimens were extracted as previously described and immediately placed in cold BIOPS solution (0 mM Ca–EGTA buffer, 0.1 M free calcium, 20 mM imidazole, 20 mM taurine, 50 mM potassium 2-[N-morpholino]-ethanesulfonic acid, 0.5 mM dithiothreitol, 6.56 mM MgCl_2_, 5.77 mM ATP, and 15 mM PCr, pH 7.1) (29). Fibers were dissected and teased apart using forceps, then permeabilized using 50 µg/mL saponin for 30 minutes. Fibers were washed with Mir05 media (0.5 mM EDTA, 3 mM MgCl_2_·6H_2_O, 60 mM K-lactobionate, 20 mM taurine, 10 mM KH_2_PO_4_, 20 mM HEPES, 110 mM sucrose, and 1 g/L BSA, pH 7.1) then dried on filter paper. The target wet weight was 2–3 mg per fiber bundle, measured using a RADWAG XA 4Y.F Analytical Balance (Radom, Poland) with a minimum load of 1 mg and readability of 0.01 mg.

### Muscle Fiber Respirometry

Ex vivo measurements of maximal complex I & II OXPHOS (Max OXPHOS) and maximal uncoupled oxygen consumption capacity (MaxETS) were taken using high-resolution respirometry in permeabilized vastus lateralis fibers on the Oroboros O2k Oxygraph system. Following the above processing procedure, fiber bundles were placed in 2 mL Mir05 media in the Oroboros O2k in duplicate. Oxygen levels were raised to 400 µM to begin. Our substrate-uncoupler-inhibitor titration protocol used 5 mM pyruvate, 2 mM malate, 4.2 mM ADP, 10 mM glutamate, and 1 µM incremental titration of carbonylcyanide-p-trifluoromethoxyphenylhydrazone (FCCP). Max OXPHOS was measured following the addition of glutamate, whereas Max ETS was measured following the addition of FCCP. All measurements were normalized to fiber bundle wet weight (pmol/s*mg). Cytochrome C was used as a quality control measure in which a greater than 15% change in oxygen consumption following Cytochrome C addition indicated compromised outer mitochondrial membrane integrity. Although there is no consensus on the exact threshold for the Cytochrome C response threshold, most agree on a range of 10%–20% (30,31).

### 31P Nuclear Magnetic Resonance Spectroscopy

Phosphorus nuclear magnetic resonance spectroscopy (31P-MRS) was used to measure in vivo maximal mitochondrial ATP production capacity (ATP Max). Participants were trained and prompted to perform isometric knee extension to promote PCr breakdown. Throughout this procedure, a 3-Tesla MR magnet (Siemen’s Medical System—Prisma Health [Pittsburgh, PA] or Skyra [Wake Forest, NC]) was used to collect phosphorus spectra through the quadriceps to quantify PCr, inorganic phosphate, and ATP levels. ATP Max was calculated as the rate of post-exercise PCr recovery, quantified as PCr level during rest divided by PCr recovery time constant ([PCr]rest/τPCr) (17,32). Detailed methodologies can be found in a previous publication (33).

### Covariate Data

Data collected included age based on self-reported date of birth, self-reported sex, and race, and self-reported ethnicity based on current census categories. Multimorbidity was classified using the SOMMA multimorbidity scale (0–11), a modification to the Rochester Epidemiology Project multimorbidity scale that includes cancer, chronic kidney disease or renal failure, atrial fibrillation, lung disease (ie, chronic obstructive pulmonary disease, bronchitis, asthma, or emphysema), coronary heart disease (ie, blocked artery or myocardial infarction), depressive symptoms, heart failure, dementia, diabetes, stroke, and aortic stenosis (34). Depressive symptoms were assessed with the 10-item version of the Center of Epidemiologic Studies Depression scale (CESD-10; score ≥10, range = 0–30) (35). Height was measured by stadiometers and weight by balance beam or digital scales. Body mass index (BMI) was then calculated as weight (kg)/height (m^2^). An approximately 6-minute-long MR scan was taken of the whole body to assess thigh muscle volume, analyzed using AMRA Researcher (AMRA Medical AB, Linköping, Sweden) by AMRA Medical (36). Physical activity was measured using the Community Healthy Activities Model Program for Seniors (CHAMPS) questionnaire, which assessed specific types and the context of physical activities (37,38). The total number of prescription medications were recorded and calculated.

### Statistical Approach

Descriptive statistics are presented as mean (*SD*) and *N* (%) and tested using *t* tests or chi-square as appropriate. We identified a significant sex × mito-modulatory medication use interaction and consequently conducted all analyses stratified by sex. Analyses performed include general linear models with marginal means (95% CI) reported. Models were adjusted for technician (for Max OXPHOS and MaxETS, 5 technicians) or site (for ATPMax, 2 sites) (M0) and further adjusted for age, race, BMI, physical activity (M1), SOMMA multimorbidity index (M2), and total medications (M3). M2 and M3 were used to test whether the effects were influenced by health status or total medication use. Additionally, inverse probability of treatment weighting (IPTW) was used as a sensitivity analysis to account for confounding by indication. Propensity scores were calculated using logistic regression and included age, race, BMI, physical activity, SOMMA morbidity index, and number of medications. Propensity score weighting was assessed using absolute standardized differences, and all factors had absolute standardized differences <0.25. IPTW models were adjusted for technician/site as appropriate. Analyses were performed using SAS 9.4 (SAS Institute, Cary, NC).

## Results

### Participant Characteristics

In this analysis, we included 448 participants with consistent medication use throughout the baseline assessment period and complete bioenergetic and covariate measurements for analyses, as depicted in Supplementary Figure S1. Participant characteristics are reported in Table 1. 57% of participants were women. The participant ages ranged from 70 to 92 years old, with a mean age of 76. In this sample of healthy older adults, 48% of participants had no multimorbidity, scoring 0 on the SOMMA multimorbidity index that comprises 11 age-related chronic conditions such as coronary heart disease, diabetes, and chronic kidney disease, among others (34). The use of one or more mito-modulatory medications was reported by 51% of participants, and 2 or more by 22% (range 0 to 4 mito-modulatory medications used).

**Table 1.** Study Sample Characteristics

	All	Men	Women	*p* Value
*N*	448	188	260	
Age (years), range	70–92	70–92	70–92	
Age (years), mean (*SD*)	76 (5)	76 (5)	76 (5)	.207
Female, *N* (%)	260 (58%)			
White, *N* (%)	394 (88%)	165 (88%)	229 (88%)	.921
Body mass index (BMI), mean (*SD*)	27.4 (4.7)	27.8 (4.3)	27.1 (4.9)	.137
Total thigh muscle volume (L), mean (*SD*)	9.1 (2.3)	11.3 (1.6)	7.5 (1.1)	<.0001
Physical activity (min/wk), mean (*SD*)	968 (631)	1 008 (702)	940 (574)	.273
SOMMA multimorbidity index, mean (*SD*)	0.71 (0.81)	0.80 (0.84)	0.64 (0.79)	.034
SOMMA multimorbidity index, *N* (%)				.072
0	215 (48%)	79 (42%)	136 (52%)	
1	164 (37%)	74 (39%)	164 (37%)	
2+	69 (15%)	35 (19%)	69 (15%)	
Total medication use, mean (*SD*)	4.0 (3.3)	4.0 (3.4)	4.1 (3.3)	.679
Total medication use, *N* (%)				.961
0–2	159 (35%)	66 (35%)	93 (36%)	
3–4	135 (30%)	58 (31%)	77 (30%)	
5+	154 (34%)	64 (34%)	90 (35%)	
Count of mito-modulatory medication use, *N* (%)				.006
0	220 (49%)	82 (44%)	138 (53%)	
1	132 (29%)	52 (28%)	80 (31%)	
2+	96 (21%)	54 (29%)	42 (16%)	
Use of 1+ mito-modulatory medication, *N* (%)	228 (51%)	106 (56%)	122 (47%)	.048
Thiazolidinedione use, *N* (%)	1 (0%)	1 (1%)	0 (0%)	.239
Biguanide use, *N* (%)	45 (10%)	31 (16%)	14 (5%)	.0001
Fluoroquinolone use, *N* (%)	1 (0%)	0 (0%)	1 (0%)	.395
Tricyclic antidepressant use, *N* (%)	3 (1%)	2 (1%)	1 (0%)	.384
Serotonin antagonist and reuptake inhibitor (SARI) use, *N* (%)	8 (2%)	1 (1%)	7 (3%)	.088
Selective serotonin and norepinephrine reuptake inhibitor (SNRI) use, *N* (%)	11 (2%)	2 (1%)	9 (3%)	.106
Atypical antidepressant use, *N* (%)	10 (2%)	5 (3%)	5 (2%)	.603
HMG CoA reductase inhibitors (statins) use, *N* (%)	168 (38%)	86 (46%)	82 (32%)	.002
Nonsteroidal antiinflammatory drugs (NSAIDs) use, *N* (%)	23 (5%)	7 (4%)	16 (6%)	.250
Salicylates use, *N* (%)	71 (16%)	35 (19%)	36 (14%)	.172
Fibrates use, *N* (%)	2 (0%)	2 (1%)	0 (0%)	.096
Max OXPHOS (pmol/(s*mg)), mean (*SD*)	62.6 (19.1)	68.3 (21.3)	58.5 (16.2)	<.0001
Max ETS (pmol/(s*mg)), mean (*SD*)	80.9 (22.5)	87.2 (24.7)	76.4 (19.7)	<.0001
ATP max (mM/time), mean (*SD*)	0.55 (0.16)	0.57 (0.17)	0.54 (0.14)	.017

*Note*: ATP = adenosine triphosphate; ETS = electron transfer system; HMG CoA = 3-hydroxy-3-methylglutaryl coenzyme A; OXPHOS = oxidative phosphorylation; SOMMA = Study of Muscle, Mobility, and Aging.

### Identification of Mito-Modulatory Medications

We defined mito-modulatory medications as medications with reported effects on mitochondria, including beneficial, harmful, and mixed effects, in the literature. In addition to a thorough literature search, we also utilized the online MitoTox database to identify medications with known mitochondrial effects (39). Mito-modulatory medications and their specific effects (eg, decreased transmembrane potential, decreased respiratory capacity, etc.) are listed in Table 2. Unadjusted participant characteristics by mito-modulatory medication use are depicted in Supplementary Table S1. We asked study participants to bring their medication bottles during each baseline visit and we collected data on medication name, formulation, route, and frequency (eg, intermittent, regular). No information regarding dosage, adherence, or indication was obtained. For this analysis, we define medication load as the number of systemic medications used.

**Table 2.** Mito-Modulatory Medications

Class	Medication(s)	Effect(s) on Mitochondria	References
Thiazolidinediones (TZD)	Pioglitazone (*n* = 1) Rosiglitazone (*n* = 0)	Decreased transmembrane potential Decreased respiratory capacity Decreased ETC complex activity Uncoupling Increased biogenesis	(1,2,40–44)
Biguanides	Metformin (*n* = 45) Buformin (*n* = 0)	Decreased transmembrane potential Dysregulated respiratory capacity Decreased ETC complex activity Decreased ATP synthesis Increased mitophagy Increased biogenesis	(3,7–9,12,13,45)
Fluoroquinolones	Ciprofloxacin (*n* = 1) Ofloxacin (*n* = 0) Levofloxacin (*n* = 0) Moxifloxacin (*n* = 0) Norfloxacin (*n* = 0)	Decreased transmembrane potential Decreased ETC complex activity Decreased ATP synthesis Dysregulation of mtDNA Increased oxidative stress	(8,46–53)
Tricyclic antidepressants	Amitriptyline (*n* = 3) Imipramine (*n* = 0) Nortriptyline (*n* = 0)	Dysregulation of respiratory capacity Decreased ETC complex activity	(54–58)
Serotonin antagonist and reuptake inhibitor (SARI)	Trazodone (*n* = 8) Nefazodone (*n* = 0)	Decreased transmembrane potential Decreased respiratory capacity Decreased ETC complex activity Decreased ATP synthesis	(3,59,60)
Selective serotonin and norepinephrine reuptake inhibitors (SNRI)	Venlafaxine (*n* = 11)	Decreased transmembrane potential Increased respiratory capacity Increased oxidative stress	(58,61)
Atypical antidepressants	Mirtazapine (*n* = 3) Bupropion (*n* = 7)	Decreased ETC complex activity Increased respiratory capacity Increased oxidative stress Increased mitochondrial volume	(62–65)
HMG CoA reductase inhibitors (statins)	Atorvastatin (*n* = 83) Lovastatin (*n* = 4) Simvastatin (*n* = 50) Rosuvastatin (*n* = 31) Pitavastatin (*n* = 0) Fluvastatin (*n* = 0)	Dysregulation of respiratory capacity Decreased ETC complex activity Decreased ATP synthesis Decreased coenzyme Q10	(4–6,66)
Nonsteroidal antiinflammatory drugs (NSAIDs)	Diclofenac (*n* = 4) Sulindac (*n* = 0) Indomethacin (*n* = 1) Meloxicam (*n* = 9) Piroxicam (*n* = 0) Naproxen (*n* = 5) Ibuprofen (*n* = 1) Celecoxib (*n* = 3)	Decreased transmembrane potential Dysregulation of respiratory capacity Decreased ETC complex activity Decreased ATP synthesis Membrane permeabilization Uncoupling	(1,67–76)
Salicylates	Acetylsalicylic acid (*n* = 71)	Increased fatty acid oxidation Increased oxidative stress Uncoupling	(70,77,78)
Fibrates	Fenofibrate (*n* = 2)	Decreased transmembrane potential Decreased ETC complex activity Uncoupling	(79–81)

*Note*: ATP = adenosine triphosphate; mtDNA = mitochondrial deoxyribonucleic acid; ETC = electron transport chain; HMG CoA = 3-hydroxy-3-methylglutaryl coenzyme A.

### Mito-Modulatory Medication Use Is Related to Lower Skeletal Muscle Bioenergetics in Men but Not Women

**Figure 1.**
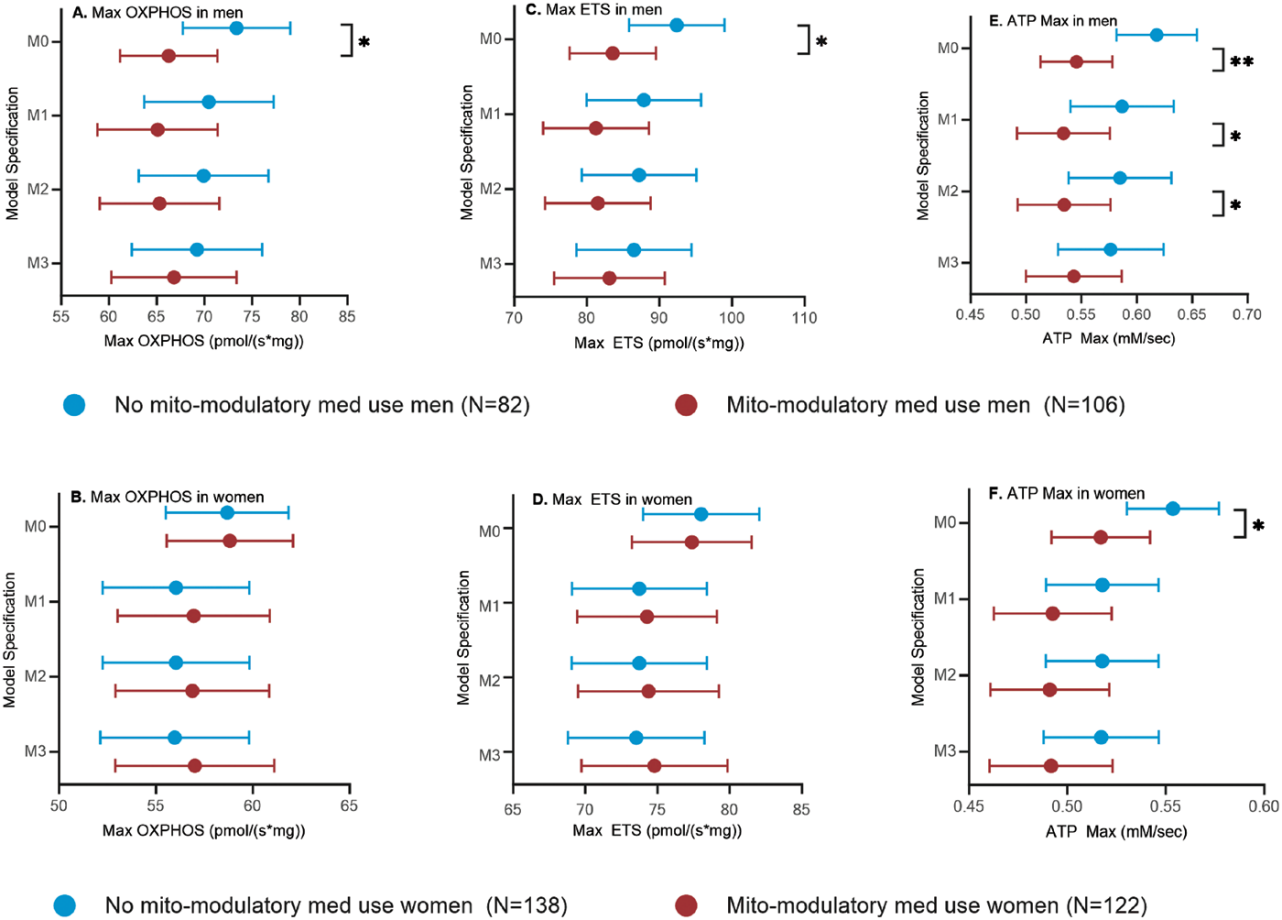
Skeletal muscle bioenergetic capacity by mito-modulatory medication use. (A) Max OXPHOS in men. (B) Max OXPHOS in women. (C) Max ETS in men. (D) Max ETS in women. (E) ATP Max in men. (F) ATP Max in women. Data are presented as marginal means (95% CI). M0: adjusted for technician (for Max OXPHOS and Max ETS) or site (for ATP Max); M1: adjusted for technician/site, age, race, BMI, physical activity; M2: adjusted for technician/site, age, race, BMI, physical activity, SOMMA multimorbidity index; M3: adjusted for technician/site, age, race, BMI, physical activity, SOMMA multimorbidity index, total medications. *p < .05, **p < .01. BMI = body mass index; SOMMA = Study of Muscle, Mobility, and Aging.

We first examined whether skeletal muscle bioenergetic capacity, measured by high-resolution respirometry to provide Max OXPHOS and Max ETS, and 31P-MRS to provide ATP Max, differed based on binary yes/no mito-modulatory medication use stratification. Sex differences were initially evaluated by including a sex × mito-modulatory medication use interaction in Model M1 including covariate adjustments for age, race, and BMI. With our a priori cut point of 0.1, interaction *p* values were generally indicative of sex differences (*p* = .029 for Max OXPHOS, *p* = .035 for Max ETS, and *p* = .32 for ATP Max) so we continued with sex-stratified analyses. Models adjusted for technician (Max OXPHOS, Max ETS) or site (ATP max) (M0) showed that mito-modulatory medication users had lower mean Max OXPHOS (66.2 pmol/(s*mg), 95% CI: 61.2–71.1) than nonusers (71.8 pmol/(s*mg), 95% CI: 67.4–78.2) and lower mean Max ETS (83.5 pmol/(s*mg), 95% CI: 77.7–83.5) than nonusers (92.2 pmol/(s*mg), 95% CI: 85.9–98.5) in men (*p* = .025, *p* = .012, respectively; Figure 1A and C, Supplementary Table S2). Similarly, mito-modulatory medication users had lower mean ATP Max (0.54 mM/s, 95% CI: 0.51–0.57) than nonusers (0.62 mM/s, 95% CI: 0.58–0.65) in men (*p* = .002; Figure 1E). Further adjustments for age, race, BMI, and physical activity (M1) and SOMMA multimorbidity index score (M2) slightly attenuated the relationships (Figure 1A, C and E, Supplementary Table S2). Fully adjusted models (M3), which also adjusted for total medication use, were not significant though the general patterns persisted (ie, were lower in users than nonusers; Figure 1A, C and E, Supplementary Table S2). We found no significant differences between mito-modulatory medication users and nonusers in women, except for ATP Max adjusted for site (users 0.51 mM/s, 95% CI: 0.49–0.54; nonusers 0.55 mM/s, 95% CI: 0.53–0.58, *p* = .028; Figure 1B, D and F, Supplementary Table S2). All adjusted models M1, M2, and M3 for women were not significant.

Sensitivity analyses indicated that total muscle volume did not significantly affect the associations between ATP Max and mito-modulatory medication use and thus was not included as a covariate in our statistical models (Supplementary Table S3).

We performed a sensitivity analysis using IPTW to account for confounding by indication. Treatment weighting analyses, depicted in Table 3, yielded similar results and confirmed the findings from our multivariate linear regression models. To demonstrate sufficient weighting, Supplementary Table S4 reports the absolute standard mean differences for each covariate before and after IPTW.

**Table 3.** Skeletal Muscle Bioenergetics by Mito-Modulatory Medication Use

Outcome	Men			Women		
	Mito-Modulatory Medication Use			Mito-Modulatory Medication Use		
	No *N* = 87	Yes *N* = 110	*p* Value	No *N* = 140	Yes *N* = 126	*p* Value
Max OXPHOS (pmol/(s*mg))	71.9 (66.0–77.7)	68.3 (62.7–73.9)	.2471	56.9 (53.8–59.9)	57.9 (54.8–61.0)	.5533
Max ETS (pmol/(s*mg))	92.8 (85.9–99.8)	86.5 (79.9–93.2)	.0911	76.5 (72.8–80.3)	78.3 (74.4–82.1)	.4607
ATP Max (mM/s)	0.59 (0.55–0.62)	0.56 (0.52–0.59)	.2305	0.54 (0.52–0.57)	0.51 (0.49–0.54)	.0720

*Note:* ATP = adenosine triphosphate; ETS = electron transfer system; OXPHOS = oxidative phosphorylation. Adjusted for age and technician (for Max OXPHOS and Max ETS) and site (for ATP Max), weighted by inverse propensity score.

### Mito-Modulatory Medication Load is Related to Lower Skeletal Muscle Bioenergetics

**Figure 2.**
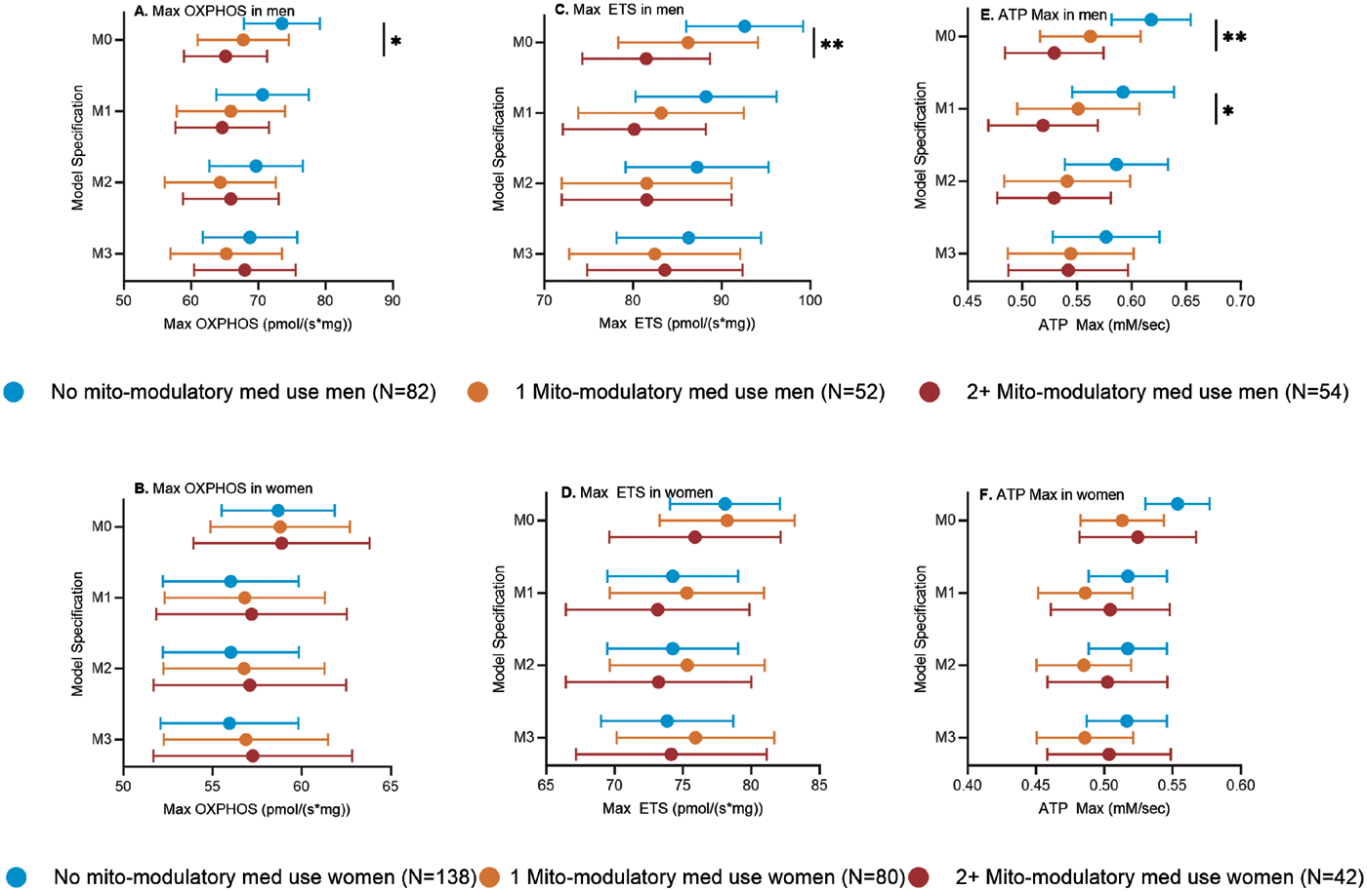
Skeletal muscle bioenergetic capacity by levels of mito-modulatory medication use. (A) Max OXPHOS in men. (B) Max OXPHOS in women. (C) Max ETS in men. (D) Max ETS in women. (E) ATP Max in men. (F) ATP Ma in women. Data are presented as marginal means (95% CI). M0: adjusted for technician (for Max OXPHOS and Max ETS) or site (for ATP Max); M1: adjusted for technician/site, age, race, BMI, physical activity; M2: adjusted for technician/site, age, race, BMI, physical activity, SOMMA multimorbidity index; M3: adjusted for technician/site, age, race, BMI, physical activity, SOMMA multimorbidity index, total medications. *p < .05, **p < .01. BMI = body mass index; SOMMA = Study of Muscle, Mobility, and Aging.

We next examined if further stratification of mito-modulatory medication use into 1 or 2+ medications would reveal an effect dependent on the number of medications. Models adjusted by technician (M0) showed a decrease in marginal means for Max OXPHOS with increasing number of medications (None 72.9 pmol/(s*mg), 95% CI: 67.5–78.3; 1 medication 67.7 pmol/(s*mg), 95% CI: 61.1–74.3; 2+ medications 65.0 pmol/(s*mg), 95% CI: 59.0–71.0; *p*-trend = .024) and for Max ETS (None 92.4 pmol/(s*mg), 95% CI: 86.1–96.7; 1 medication 86.3 pmol/(s*mg), 95% CI: 78.6–93.9; 2+ medications 81.3 pmol/(s*mg), 95% CI: 74.3–88.3; *p*-trend = .007) in men (Figure 2A and C, Supplementary Table S5). Similarly, models adjusted for site (M0) showed a decrease in marginal means of ATP Max with increasing number of medications (None 0.62 mM/s, 95% CI: 0.58–0.65; 1 medication 0.56 mM/s, 95% CI: 0.52–0.61; 2+ medications 0.52 mM/s, 95% CI: 0.48–0.57; *p*-trend = .001) in men (Figure 2E, Supplementary Table S5). Adjustments for covariates also attenuated the associations. We found no significant differences in any models for women (Figure 2B, D and F, Supplementary Table S5).

## Discussion

To our knowledge, this is the first study to examine the relationship between burden of mito-modulatory medication use and skeletal muscle bioenergetics in older adults. Use of one or more mito-modulatory medications was associated with lower skeletal muscle bioenergetics for men but not for women. Men using mito-modulatory medications had lower levels of 3 measures of mitochondrial bioenergetic capacity, Max OXPHOS, and Max ETS from ex vivo high-resolution respirometry and ATP Max from in vivo 31P-MRS, compared to nonusers, with little to no difference in users versus nonusers in women.

The present study builds upon our incomplete understanding of the potential impacts of mito-modulatory medication use in humans. To date, studies have generally focused on one specific medication or medication class in a small sample. For example, Konopka et al. examined the effect of metformin on skeletal muscle respiration and mitochondrial adaptations to exercise in 53 healthy participants and found that metformin attenuated the positive changes in mitochondrial respiration associated with exercise recovery (82). Additionally, there is substantial interest in the effect of statins (eg, simvastatin) given their widely recognized effects on skeletal muscle metabolism. Results have been mixed. A study by van Diemen et al. used 31P-MRS and found that 4 weeks of simvastatin treatment reduced ATP production in 28 healthy participants (83). This is in line with the LIFESTAT study, which found that statin use was related to reduced skeletal muscle complex II-linked respiration in 39 participants (84). However, the same study found that statin-induced myalgia was coupled to an increased intrinsic mitochondrial respiratory function in 25 participants. A study of 40 patients by Durhuus et al. found that taking simvastatin for over 6 months resulted in higher blood cell bioenergetic capacity compared to nonuser controls (85). These mixed results highlight the complexity of the impacts of mito-modulatory medication use. Our focus on medication load, rather than individual medications, provides new insights that complement studies that have focused on individual medications. Our findings align with the premise that higher medication burden, or number of medications, is related to more adverse or unwanted effects. Importantly, the interaction between mito-modulatory medications and skeletal muscle bioenergetics may help explain drug-induced impairments in exercise adaptations. Others have reported that some medications, including metformin, statins, and nonsteroidal antiinflammatory drugs (NSAIDs), exert detrimental effects on adaptations to exercise and that these detriments might be explained through a mitochondrial mechanism (86–88). Here, we report that those same medications may contribute to lower skeletal muscle bioenergetics in men, providing a potential link between medication use and impaired exercise adaptations.

Interestingly, we observed significant associations in men but not in women. The significant interaction between sex and mito-modulatory medication use suggests a true difference, rather than a lack of statistical power, between men and women. There are a few reasons that may explain this sex disparity. Inherent differences in mitochondrial function and quality between men and women have been previously reported by our group and others. A recent analysis of sex-based differences in SOMMA participants found that women had lower skeletal muscle bioenergetics compared to men, possibly contributing to the disparity in mobility impairment between men and women (29). Other groups such as Cardinale et al., found significant differences in intrinsic mitochondrial respiration (respiration per amount of mitochondrial protein) and oxygen affinity between men and women, which implied varied adaptations to oxygen delivery (89). Similarly, Scalzo et al. found that mitochondrial protein synthesis was higher in men compared to women after exercise (90). The differences in mitochondrial bioenergetic function between men and women could factor into their response to medications that may modulate it. Further, there are well-known sex-based differences in drug kinetics and metabolism (91). Put together, it is unsurprising that mito-modulatory medication use can confer different outcomes in skeletal muscle in men compared to skeletal muscle in women.

It is important to consider that our findings may be driven by underlying health status or disease rather than by medication load, especially considering that the “total medications” covariate attenuated the observed association of our statistical model. To address this confounding by indication, we conducted an IPTW analysis, which is commonly used to control for the influence of confounders in statistical inference (92). We found similar significant results in our IPTW models that are in line with our multivariate linear regression models, suggesting that the effect of medication load persists without the influence of total medication use or multimorbidity score. We can surmise that the associations between mito-modulatory medication use and lower skeletal muscle bioenergetics are likely from the medication load itself rather than underlying or confounding health factors.

Limitations of this study include the lack of electronic health records for participant medical history. All medication use data were obtained by pill container examination, but no information regarding dosage, adherence, or indication was obtained. Only data on prescription medication use was recorded, so use of over-the-counter medications (eg, NSAIDs) may be underreported. This would bias our results toward the null. Additionally, the study population was relatively healthy (48% with multimorbidity score of 0) for a study of medication use and polypharmacy. Two of the 11 mito-modulatory medication classes, statins and biguanides, had a statistically significant difference in the number of men and women taking them, potentially contributing to the different outcomes in men and women. However, the absolute number of users (ie, 86 men and 82 women for statins, 31 men and 14 women for biguanides) reveals that the difference may not be substantial, especially considering the total *N* of 188 men and 260 women. Another limitation is that, though we utilize 2 separate but complementary measures of skeletal muscle bioenergetics, we lack measures of other skeletal muscle mitochondrial properties such as ROS handling, calcium dynamics, or fatty acid utilization. As with all retrospective cohort studies, our results do not demonstrate causality but rather an association. Since observational analyses of medication use are subject to confounding by indication, we cannot rule out that our results reflect unresolved confounding. We acknowledge that some health conditions may be related to mitochondrial dysfunction and cannot be fully accounted for (eg, diabetes). However, we undertook 2 approaches to account for confounding (adjusting for potential confounders in linear regression models and IPTW).

In conclusion, this study provides novel insight into the potential consequences of using mito-modulatory medications. Our findings suggest that many older adults are taking a variety of medications that appear to elicit a detrimental effect on mitochondrial function reflected by worse skeletal muscle bioenergetic capacity. It is noteworthy that these potential adverse effects are not captured in clinical trials or in the drug labels, thus largely unknown. Our study highlights the importance and need to rigorously test and document these mitochondrial effects with specific medications in randomized settings to establish causality and better inform patients and prescribers of the potential clinical impact of using these medications. Based on the results presented here, future studies examining the impact of medication use on functional outcomes (eg, strength, fitness) should consider mitochondrial bioenergetics as a potential mediator.

## Supplementary Material

glaf063_suppl_Supplementary_Materials

## Data Availability

The analysis data set for this specific manuscript is available on request from the corresponding author. The SOMMA data are available to the public: https://sommaonline.ucsf.edu.

## Code Availability

Computer code is also available on request from the corresponding author.

## References

[1] Nadanaciva S, Rana P, Beeson GC, et al Assessment of drug-induced mitochondrial dysfunction via altered cellular respiration and acidification measured in a 96-well platform. J Bioenerg Biomembr. 2012;44(4):421–437. https://doi.org/10.1007/s10863-012-9446-z 22689143

[2] Eakins J, Bauch C, Woodhouse H, et al A combined in vitro approach to improve the prediction of mitochondrial toxicants. Toxicol Vitro Int J Publ Assoc BIBRA. 2016;34:161–170. https://doi.org/10.1016/j.tiv.2016.03.016 27083147

[3] Nadanaciva S, Bernal A, Aggeler R, et al Target identification of drug induced mitochondrial toxicity using immunocapture based OXPHOS activity assays. Toxicol Vitro Int J Publ Assoc BIBRA. 2007;21(5):902–911. https://doi.org/10.1016/j.tiv.2007.01.011 17346924

[4] Apostolopoulou M, Corsini A, Roden M. The role of mitochondria in statin-induced myopathy. Eur J Clin Invest. 2015;45(7):745–754. https://doi.org/10.1111/eci.12461 25991405

[5] Kaufmann P, Török M, Zahno A, et al Toxicity of statins on rat skeletal muscle mitochondria. Cell Mol Life Sci. 2006;63(19–20):2415–2425. https://doi.org/10.1007/s00018-006-6235-z 17013560 PMC11136287

[6] Mullen PJ, Zahno A, Lindinger P, et al Susceptibility to simvastatin-induced toxicity is partly determined by mitochondrial respiration and phosphorylation state of Akt. Biochim Biophys Acta. 2011;1813(12):2079–2087. https://doi.org/10.1016/j.bbamcr.2011.07.019 21839782

[7] Dykens JA, Jamieson J, Marroquin L, et al Biguanide-induced mitochondrial dysfunction yields increased lactate production and cytotoxicity of aerobically-poised HepG2 cells and human hepatocytes in vitro. Toxicol Appl Pharmacol. 2008;233(2):203–210. https://doi.org/10.1016/j.taap.2008.08.013 18817800

[8] Porceddu M, Buron N, Roussel C, et al Prediction of liver injury induced by chemicals in human with a multiparametric assay on isolated mouse liver mitochondria. Toxicol Sci. 2012;129(2):332–345. https://doi.org/10.1093/toxsci/kfs197 22987451 PMC3446843

[9] Andrzejewski S, Gravel SP, Pollak M, et al Metformin directly acts on mitochondria to alter cellular bioenergetics. Cancer Metab. 2014;2:12. https://doi.org/10.1186/2049-3002-2-12 25184038 PMC4147388

[10] Kane S. ClinCalc DrugStats Database. https://clincalc.com/DrugStats/. Accessed January 29, 2025.

[11] Stoker ML, Newport E, Hulit JC, et al Impact of pharmacological agents on mitochondrial function: a growing opportunity? Biochem Soc Trans. 2019;47(6):1757–1772. https://doi.org/10.1042/BST20190280 31696924 PMC6925523

[12] de Marañón AM, Díaz-Pozo P, Canet F, et al Metformin modulates mitochondrial function and mitophagy in peripheral blood mononuclear cells from type 2 diabetic patients. Redox Biol. 2022;53:102342. https://doi.org/10.1016/j.redox.2022.102342 35605453 PMC9124713

[13] Bhansali S, Bhansali A, Dutta P, et al Metformin upregulates mitophagy in patients with T2DM: a randomized placebo‐controlled study. J Cell Mol Med. 2020;24(5):2832–2846. https://doi.org/10.1111/jcmm.14834 31975558 PMC7077543

[14] Young EH, Pan S, Yap AG, et al Polypharmacy prevalence in older adults seen in United States physician offices from 2009 to 2016. PLoS One. 2021;16(8):e0255642. https://doi.org/10.1371/journal.pone.0255642 34343225 PMC8330900

[15] Qato DM, Wilder J, Schumm LP, et al Changes in prescription and over-the-counter medication and dietary supplement use among older adults in the United States, 2005 vs 2011. JAMA Intern Med. 2016;176(4):473–482. https://doi.org/10.1001/jamainternmed.2015.8581 26998708 PMC5024734

[16] Nadanaciva S, Dykens JA, Bernal A, et al Mitochondrial impairment by PPAR agonists and statins identified via immunocaptured OXPHOS complex activities and respiration. Toxicol Appl Pharmacol. 2007;223(3):277–287. https://doi.org/10.1016/j.taap.2007.06.003 17658574

[17] Coen PM, Jubrias SA, Distefano G, et al Skeletal muscle mitochondrial energetics are associated with maximal aerobic capacity and walking speed in older adults. J Gerontol A Biol Sci Med Sci. 2013;68(4):447–455. https://doi.org/10.1093/gerona/gls196 23051977 PMC3593613

[18] Tyrrell DJ, Bharadwaj MS, Van Horn CG, et al Respirometric profiling of muscle mitochondria and blood cells are associated with differences in gait speed among community-dwelling older adults. J Gerontol A Biol Sci Med Sci. 2015;70(11):1394–1399. https://doi.org/10.1093/gerona/glu096 25030980 PMC4731403

[19] Short KR, Bigelow ML, Kahl J, et al Decline in skeletal muscle mitochondrial function with aging in humans. Proc Natl Acad Sci U S A. 2005;102(15):5618–5623. https://doi.org/10.1073/pnas.0501559102 15800038 PMC556267

[20] Grevendonk L, Connell NJ, McCrum C, et al Impact of aging and exercise on skeletal muscle mitochondrial capacity, energy metabolism, and physical function. Nat Commun. 2021;12(1):4773. https://doi.org/10.1038/s41467-021-24956-2 34362885 PMC8346468

[21] Conley KE, Jubrias SA, Esselman PC. Oxidative capacity and ageing in human muscle. J Physiol. 2000;526 Pt 1(Pt 1):203–210. https://doi.org/10.1111/j.1469-7793.2000.t01-1-00203.x 10878112 PMC2269983

[22] Joseph A-M, Adhihetty PJ, Buford TW, et al The impact of aging on mitochondrial function and biogenesis pathways in skeletal muscle of sedentary high- and low-functioning elderly individuals. Aging Cell. 2012;11(5):801–809. https://doi.org/10.1111/j.1474-9726.2012.00844.x 22681576 PMC3444680

[23] Schunk K, Pitton M, Düber C, et al Dynamic phosphorus-31 magnetic resonance spectroscopy of the quadriceps muscle: effects of age and sex on spectroscopic results. Invest Radiol. 1999;34(2):116–125. https://doi.org/10.1097/00004424-199902000-00004 9951791

[24] Cummings SR, Newman AB, Coen PM, et al The Study of Muscle, Mobility and Aging (SOMMA): a unique cohort study about the cellular biology of aging and age-related loss of mobility. J Gerontol A Biol Sci Med Sci. 2023;78(11):2083–2093. https://doi.org/10.1093/gerona/glad052 36754371 PMC10613002

[25] Nelson SJ, Zeng K, Kilbourne J, et al Normalized names for clinical drugs: RxNorm at 6 years. J Am Med Inform Assoc. 2011;18(4):441–448. https://doi.org/10.1136/amiajnl-2011-000116 21515544 PMC3128404

[26] Fung KW, McDonald C, Bray BE. RxTerms - a drug interface terminology derived from RxNorm. AMIA Annu Symp Proc. 2008;2008:227–231. 18998891 PMC2655997

[27] Bodenreider O, Peters LB. RxMis—Use of NLM drug APIs by non-programmers. *AMIA.* November 2017.

[28] Lagerlund O, Strese S, Fladvad M, et al A global, validated and updated dictionary for medicinal information. Ther Innov Regul Sci. 2020;54(5):1116–1122. https://doi.org/10.1007/s43441-020-00130-6 32078733 PMC7458889

[29] Kramer PA, Coen PM, Cawthon PM, et al skeletal muscle energetics explain the sex disparity in mobility impairment in the Study of Muscle, Mobility and Aging. J Gerontol A Biol Sci Med Sci. 2024;79(4):glad283. https://doi.org/10.1093/gerona/glad283 38150179 PMC10960628

[30] Kuang J, Saner NJ, Botella J, et al Assessing mitochondrial respiration in permeabilized fibres and biomarkers for mitochondrial content in human skeletal muscle. Acta Physiol. 2022;234(2):e13772. https://doi.org/10.1111/apha.13772 34985815

[31] Krajčová A, Urban T, Megvinet D, et al High resolution respirometry to assess function of mitochondria in native homogenates of human heart muscle. PLoS One. 2020;15(1):e0226142. https://doi.org/10.1371/journal.pone.0226142 31940313 PMC6961865

[32] Amara CE, Marcinek DJ, Shankland EG, et al Mitochondrial function in vivo: spectroscopy provides window on cellular energetics. Methods San Diego Calif. 2008;46(4):312–318. https://doi.org/10.1016/j.ymeth.2008.10.001 18930151 PMC10798296

[33] Mau T, Lui L-Y, Distefano G, et al Mitochondrial energetics in skeletal muscle are associated with leg power and cardiorespiratory fitness in the Study of Muscle, Mobility and Aging. J Gerontol A Biol Sci Med Sci. 2023;78(8):1367–1375. https://doi.org/10.1093/gerona/glac238 36462195 PMC10395564

[34] Mau T, Blackwell TL, Cawthon PM, et al Muscle mitochondrial bioenergetic capacities is associated with multimorbidity burden in older adults: the Study of Muscle, Mobility and Aging (SOMMA). *J Gerontol A Biol Sci Med Sci*. 2024;79(7):glae101. https://doi.org/10.1101/2023.11.06.23298175 PMC11167490 38605684

[35] Irwin M, Artin KH, Oxman MN. Screening for depression in the older adult: criterion validity of the 10-item Center for Epidemiological Studies Depression scale (CES-D). Arch Intern Med. 1999;159(15):1701–1704. https://doi.org/10.1001/archinte.159.15.1701 10448771

[36] Romu T, Borga M, Dahlqvist O. MANA—Multi scale adaptive normalized averaging. 2011 IEEE International Symposium on Biomedical Imaging: From Nano to Macro, Chicago, IL, 2011:361–364. https://doi.org/10.1109/isbi.2011.5872424

[37] Hekler EB, Buman MP, Haskell WL, et al Reliability and validity of CHAMPS self-reported sedentary-to-vigorous intensity physical activity in older adults. J Phys Act Health. 2012;9(2):225–236. https://doi.org/10.1123/jpah.9.2.225 22368222 PMC4733646

[38] Stewart AL, Mills KM, King AC, et al CHAMPS physical activity questionnaire for older adults: outcomes for interventions. Med Sci Sports Exerc. 2001;33(7):1126–1141. https://doi.org/10.1097/00005768-200107000-00010 11445760

[39] Lin Y-T, Lin K-H, Huang C-J, et al A comprehensive mitochondrial toxicity database. BMC Bioinf. 2021;22(Suppl 10):369. https://doi.org/10.1186/s12859-021-04285-3 PMC8283953 34266386

[40] Masubuchi Y, Kano S, Horie T. Mitochondrial permeability transition as a potential determinant of hepatotoxicity of antidiabetic thiazolidinediones. Toxicology. 2006;222(3):233–239. https://doi.org/10.1016/j.tox.2006.02.017 16621215

[41] Segawa M, Sekine S, Sato T, et al Increased susceptibility to troglitazone-induced mitochondrial permeability transition in type 2 diabetes mellitus model rat. J Toxicol Sci. 2018;43(5):339–351. https://doi.org/10.2131/jts.43.339 29743445

[42] Riess ML, Elorbany R, Weihrauch D, et al PPARγ-independent side effects of thiazolidinediones on mitochondrial redox state in rat isolated hearts. Cells. 2020;9(1):252. https://doi.org/10.3390/cells9010252 31968546 PMC7017211

[43] Rong JX, Klein J-LD, Qiu Y, et al Rosiglitazone induces mitochondrial biogenesis in differentiated murine 3T3-L1 and C3H/10T1/2 adipocytes. PPAR Res. 2011;2011:179454. https://doi.org/10.1155/2011/179454 22013433 PMC3195302

[44] Pardo R, Enguix N, Lasheras J, et al Rosiglitazone-induced mitochondrial biogenesis in white adipose tissue is independent of peroxisome proliferator-activated receptor γ coactivator-1α. PLoS One. 2011;6(11):e26989. https://doi.org/10.1371/journal.pone.0026989 22087241 PMC3210129

[45] Feng J, Wang X, Ye X, et al Mitochondria as an important target of metformin: the mechanism of action, toxic and side effects, and new therapeutic applications. Pharmacol Res. 2022;177:106114. https://doi.org/10.1016/j.phrs.2022.106114 35124206

[46] Hangas A, Aasumets K, Kekäläinen NJ, et al Ciprofloxacin impairs mitochondrial DNA replication initiation through inhibition of Topoisomerase 2. Nucleic Acids Res. 2018;46(18):9625–9636. https://doi.org/10.1093/nar/gky793 30169847 PMC6182158

[47] Papa V, Leonardi A, Getuli C, et al Effect of ofloxacin and netilmicin on human corneal and conjunctival cells in vitro. J Ocul Pharmacol Ther. 2003;19(6):535–545. https://doi.org/10.1089/108076803322660459 14733711

[48] Ghaly H, Jörns A, Rustenbeck I. Effect of fluoroquinolones on mitochondrial function in pancreatic beta cells. Eur J Pharm Sci. 2014;52:206–214. https://doi.org/10.1016/j.ejps.2013.11.011 24284031

[49] Song M, Wu H, Wu S, et al Antibiotic drug levofloxacin inhibits proliferation and induces apoptosis of lung cancer cells through inducing mitochondrial dysfunction and oxidative damage. Biomed Pharmacother. 2016;84:1137–1143. https://doi.org/10.1016/j.biopha.2016.10.034 27780143

[50] Lawrence JW, Claire DC, Weissig V, et al Delayed cytotoxicity and cleavage of mitochondrial DNA in ciprofloxacin-treated mammalian cells. Mol Pharmacol. 1996;50(5):1178–1188. 8913349

[51] Lowes DA, Wallace C, Murphy MP, et al The mitochondria targeted antioxidant MitoQ protects against fluoroquinolone-induced oxidative stress and mitochondrial membrane damage in human Achilles tendon cells. Free Radic Res. 2009;43(4):323–328. https://doi.org/10.1080/10715760902736275 19235604

[52] Pouzaud F, Bernard-Beaubois K, Thevenin M, et al In vitro discrimination of fluoroquinolones toxicity on tendon cells: involvement of oxidative stress. J Pharmacol Exp Ther. 2004;308(1):394–402. https://doi.org/10.1124/jpet.103.057984 14569066

[53] Sheng Z, Cao X, Peng S, et al Ofloxacin induces apoptosis in microencapsulated juvenile rabbit chondrocytes by caspase-8-dependent mitochondrial pathway. Toxicol Appl Pharmacol. 2008;226(2):119–127. https://doi.org/10.1016/j.taap.2007.08.025 17961619

[54] Hroudová J, Fišar Z. In vitro inhibition of mitochondrial respiratory rate by antidepressants. Toxicol Lett. 2012;213(3):345–352. https://doi.org/10.1016/j.toxlet.2012.07.017 22842584

[55] Réus GZ, Stringari RB, Rezin GT, et al Administration of memantine and imipramine alters mitochondrial respiratory chain and creatine kinase activities in rat brain. J Neural Transm Vienna Austria 1996. 2012;119(4):481–491. https://doi.org/10.1007/s00702-011-0718-2 21953515

[56] Katyare SS, Rajan RR. Effect of long-term in vivo treatment with imipramine on the oxidative energy metabolism in rat brain mitochondria. Comp Biochem Physiol C Pharmacol Toxicol Endocrinol. 1995;112(3):353–357. https://doi.org/10.1016/0742-8413(95)02031-4 8838689

[57] Eto K, Fukuda T, Araki Y, et al Effect of tricyclic drugs on mitochondrial membrane. Acta Med Okayama. 1985;39(4):289–295. https://doi.org/10.18926/AMO/31500 2931948

[58] Scaini G, Maggi DD, De-Nês BT, et al Activity of mitochondrial respiratory chain is increased by chronic administration of antidepressants. Acta Neuropsychiatr. 2011;23(3):112–118. https://doi.org/10.1111/j.1601-5215.2011.00548.x 26952897

[59] Dykens JA, Jamieson JD, Marroquin LD, et al In vitro assessment of mitochondrial dysfunction and cytotoxicity of nefazodone, trazodone, and buspirone. Toxicol Sci. 2008;103(2):335–345. https://doi.org/10.1093/toxsci/kfn056 18344530

[60] Taziki S, Sattari MR, Eghbal MA. Mechanisms of trazodone-induced cytotoxicity and the protective effects of melatonin and/or taurine toward freshly isolated rat hepatocytes. J Biochem Mol Toxicol. 2013;27(10):457–462. https://doi.org/10.1002/jbt.21509 24023050

[61] Ahmadian E, Babaei H, Mohajjel Nayebi A, et al Venlafaxine-induced cytotoxicity towards isolated rat hepatocytes involves oxidative stress and mitochondrial/lysosomal dysfunction. Adv Pharm Bull. 2016;6(4):521–530. https://doi.org/10.15171/apb.2016.066 28101459 PMC5241410

[62] Bielecka-Wajdman AM, Ludyga T, Machnik G, et al Tricyclic antidepressants modulate stressed mitochondria in glioblastoma multiforme cells. Cancer Control. 2018;25(1):1073274818798594. https://doi.org/10.1177/1073274818798594 30213208 PMC6144521

[63] Hroudová J, Fišar Z. Connectivity between mitochondrial functions and psychiatric disorders. Psychiatry Clin Neurosci. 2011;65(2):130–141. https://doi.org/10.1111/j.1440-1819.2010.02178.x 21414088

[64] Jang E-H, Park C-S, Kang J-H. Bupropion, an atypical antidepressant, induces endoplasmic reticulum stress and caspase-dependent cytotoxicity in SH-SY5Y cells. Toxicology. 2011;285(1-2):1–7. https://doi.org/10.1016/j.tox.2011.02.006 21354251

[65] Ferreira GK, Rezin GT, Cardoso MR, et al Brain energy metabolism is increased by chronic administration of bupropion. Acta Neuropsychiatr. 2012;24(2):115–121. https://doi.org/10.1111/j.1601-5215.2011.00597.x 26952953

[66] Vaughan RA, Garcia-Smith R, Bisoffi M, et al Ubiquinol rescues simvastatin-suppression of mitochondrial content, function and metabolism: implications for statin-induced rhabdomyolysis. Eur J Pharmacol. 2013;711(1-3):1–9. https://doi.org/10.1016/j.ejphar.2013.04.009 23624330

[67] Pritchard R, Rodríguez-Enríquez S, Pacheco-Velázquez SC, et al Celecoxib inhibits mitochondrial O2 consumption, promoting ROS dependent death of murine and human metastatic cancer cells via the apoptotic signalling pathway. Biochem Pharmacol. 2018;154:318–334. https://doi.org/10.1016/j.bcp.2018.05.013 29800556

[68] Atashbar S, Jamali Z, Khezri S, et al Celecoxib decreases mitochondrial complex IV activity and induces oxidative stress in isolated rat heart mitochondria: an analysis for its cardiotoxic adverse effect. J Biochem Mol Toxicol. 2022;36(1):e22934. https://doi.org/10.1002/jbt.22934 34668290

[69] Syed M, Skonberg C, Hansen SH. Mitochondrial toxicity of diclofenac and its metabolites via inhibition of oxidative phosphorylation (ATP synthesis) in rat liver mitochondria: possible role in drug induced liver injury (DILI). *Toxicol Vitro Int J Publ Assoc BIBRA*. Toxicol In Vitro. 2016;31:93–102. https://doi.org/10.1016/j.tiv.2015.11.020 26627130

[70] Petrescu I, Tarba C. Uncoupling effects of diclofenac and aspirin in the perfused liver and isolated hepatic mitochondria of rat. Biochim Biophys Acta. 1997;1318(3):385–394. https://doi.org/10.1016/s0005-2728(96)00109-0 9048975

[71] Moreno-Sánchez R, Bravo C, Vásquez C, et al Inhibition and uncoupling of oxidative phosphorylation by nonsteroidal anti-inflammatory drugs: study in mitochondria, submitochondrial particles, cells, and whole heart. Biochem Pharmacol. 1999;57(7):743–752. https://doi.org/10.1016/s0006-2952(98)00330-x 10075080

[72] Shao D, Kan M, Qiao P, et al Celecoxib induces apoptosis via a mitochondria‑dependent pathway in the H22 mouse hepatoma cell line. Mol Med Rep. 2014;10(4):2093–2098. https://doi.org/10.3892/mmr.2014.2461 25109418

[73] Chakraborty H, Chakraborty PK, Raha S, et al Interaction of piroxicam with mitochondrial membrane and cytochrome c. Biochim Biophys Acta. 2007;1768(5):1138–1146. https://doi.org/10.1016/j.bbamem.2007.01.004 17306218

[74] Brandolini L, Antonosante A, Giorgio C, et al NSAIDs-dependent adaption of the mitochondria-proteasome system in immortalized human cardiomyocytes. Sci Rep. 2020;10(1):18337. https://doi.org/10.1038/s41598-020-75394-x 33110169 PMC7591859

[75] Somasundaram S, Rafi S, Hayllar J, et al Mitochondrial damage: a possible mechanism of the “topical” phase of NSAID induced injury to the rat intestine. Gut. 1997;41(3):344–353. https://doi.org/10.1136/gut.41.3.344 9378390 PMC1891477

[76] Leite S, Martins NM, Dorta DJ, et al Mitochondrial uncoupling by the sulindac metabolite, sulindac sulfide. Basic Clin Pharmacol Toxicol. 2006;99(4):294–299. https://doi.org/10.1111/j.1742-7843.2006.pto_490.x 17040214

[77] Uppala R, Dudiak B, Beck ME, et al Aspirin increases mitochondrial fatty acid oxidation. Biochem Biophys Res Commun. 2017;482(2):346–351. https://doi.org/10.1016/j.bbrc.2016.11.066 27856258 PMC5195905

[78] Raza H, John A. Implications of altered glutathione metabolism in aspirin-induced oxidative stress and mitochondrial dysfunction in HepG2 cells. PLoS One. 2012;7(4):e36325. https://doi.org/10.1371/journal.pone.0036325 22558435 PMC3340360

[79] Brunmair B, Lest A, Staniek K, et al Fenofibrate impairs rat mitochondrial function by inhibition of respiratory complex I. J Pharmacol Exp Ther. 2004;311(1):109–114. https://doi.org/10.1124/jpet.104.068312 15166256

[80] Yamada K, Tsunoda K, Kawai K, et al Mitochondria toxicity of antihyperlipidemic agents bezafibrate and fenofibrate. Diabetol Int. 2013;4(2):126–131. https://doi.org/10.1007/s13340-012-0104-9

[81] Qu B, Li QT, Wong KP, et al Mechanism of clofibrate hepatotoxicity: mitochondrial damage and oxidative stress in hepatocytes. Free Radic Biol Med. 2001;31(5):659–669. https://doi.org/10.1016/s0891-5849(01)00632-3 11522451

[82] Konopka AR, Laurin JL, Schoenberg HM, et al Metformin inhibits mitochondrial adaptations to aerobic exercise training in older adults. Aging Cell. 2019;18(1):e12880. https://doi.org/10.1111/acel.12880 30548390 PMC6351883

[83] van Diemen MPJ, Berends CL, Akram N, et al Validation of a pharmacological model for mitochondrial dysfunction in healthy subjects using simvastatin: a randomized placebo-controlled proof-of-pharmacology study. Eur J Pharmacol. 2017;815:290–297. https://doi.org/10.1016/j.ejphar.2017.09.031 28943100

[84] Dohlmann TL, Morville T, Kuhlman AB, et al Statin treatment decreases mitochondrial respiration but muscle coenzyme Q10 levels are unaltered: the LIFESTAT study. J Clin Endocrinol Metab. 2019;104(7):2501–2508. https://doi.org/10.1210/jc.2018-01185 30299473

[85] Durhuus JA, Hansson S, Morville T, et al Simvastatin improves mitochondrial respiration in peripheral blood cells. Sci Rep. 2020;10(1):17012. https://doi.org/10.1038/s41598-020-73896-2 33046789 PMC7550337

[86] Mikus CR, Boyle LJ, Borengasser SJ, et al Simvastatin impairs exercise training adaptations. J Am Coll Cardiol. 2013;62(8):709–714. https://doi.org/10.1016/j.jacc.2013.02.074 23583255 PMC3745788

[87] Robinson MM, Hamilton KL, Miller BF. The interactions of some commonly consumed drugs with mitochondrial adaptations to exercise. J Appl Physiol (1985). 2009;107(1):8–16. https://doi.org/10.1152/japplphysiol.00343.2009 19423832

[88] Miller BF, Thyfault JP. Exercise-pharmacology interactions: metformin, statins, and healthspan. Physiology (Bethesda). 2020;35(5):338–347. https://doi.org/10.1152/physiol.00013.2020 32783612 PMC7642846

[89] Cardinale DA, Larsen FJ, Schiffer TA, et al Superior Intrinsic Mitochondrial Respiration in Women than in Men. Front Physiol. 2018;9:1133. https://doi.org/10.3389/fphys.2018.01133 30174617 PMC6108574

[90] Scalzo RL, Peltonen GL, Binns SE, et al Greater muscle protein synthesis and mitochondrial biogenesis in males compared with females during sprint interval training. FASEB J. 2014;28(6):2705–2714. https://doi.org/10.1096/fj.13-246595 24599968

[91] Soldin O, Mattison D. Sex differences in pharmacokinetics and pharmacodynamics. Clin Pharmacokinet. 2009;48(3):143–157. https://doi.org/10.2165/00003088-200948030-00001 19385708 PMC3644551

[92] Chesnaye NC, Stel VS, Tripepi G, et al An introduction to inverse probability of treatment weighting in observational research. Clin Kidney J. 2021;15(1):14–20. https://doi.org/10.1093/ckj/sfab158 35035932 PMC8757413

