# Supplementary material for: Mito-Modulatory Medication Use and Skeletal Muscle Bioenergetics Among Older Men and Women: The Study of Muscle, Mobility, and Aging
Source: J Gerontol A Biol Sci Med Sci. 2025 Mar 23;80(6):glaf063. doi: 10.1093/gerona/glaf063 (PMC12128851; doi:10.1093/gerona/glaf063)
Supplement: glaf063_suppl_Supplementary_Materials [file glaf063_suppl_supplementary_materials.pdf]

List of Supplementary Materials

eFigure 1. Flow diagram of sample selection.

eTable 1. Study sample characteristics by mito-modulatory medication use.

eTable 2: Skeletal muscle bioenergetic capacity by mito-modulatory medication use.

eTable 3: ATP Max by mito-modulatory medication use.

eTable 4: Absolute standardized mean difference before and after inverse probability of treatment weighting analysis.

eTable 5: Skeletal muscle bioenergetic capacity by levels of mito-modulatory medication use.

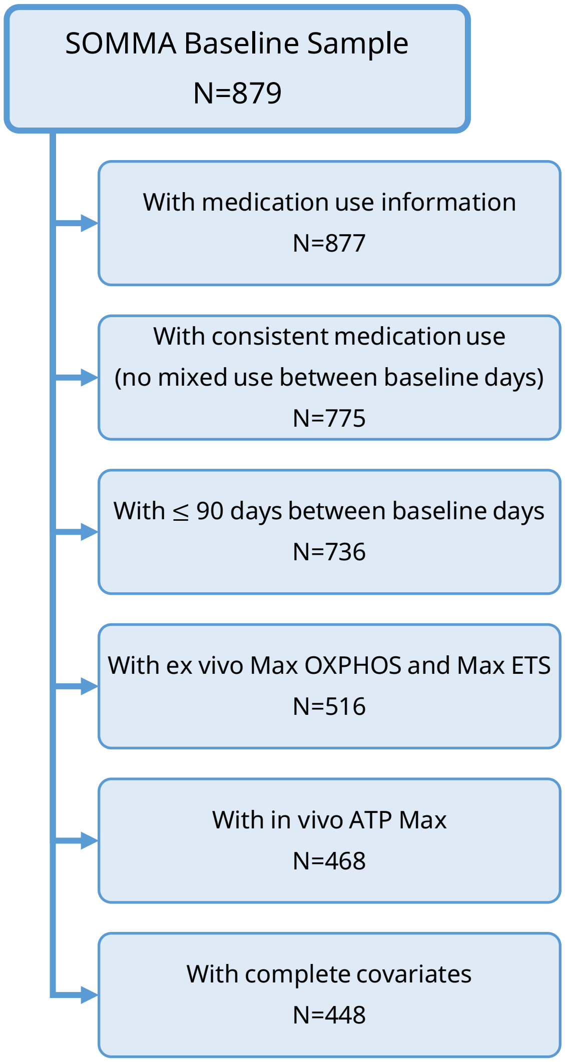

eFigure 1. Flow diagram of sample selection. Participants were included if they had consistent medication use, all outcomes of interest, and complete covariates. SOMMA = Study of Muscle, Mobility and Aging; Max OXPHOS = maximal complex I&II supported OXPHOS; Max ETS = maximal uncoupled respiration; ATP Max = maximal mitochondrial adenosine triphosphate production.

eTable 1. Study sample characteristics by mito-modulatory medication use.

	Mito-Modulatory Medication Use					
	Men			Women		
	No N=82	Yes N=106	p-value	No N=138	Yes N=122	p-value
Age (years), range	70-92	70-91		70-90	70-92	
Age (years), mean (SD)	75 (4)	76 (5)	.039	76 (5)	76 (5)	.851
White, N (%)	73 (89%)	92 (87%)	.643	123 (89%)	106 (87%)	.577
Body mass index (BMI), mean (SD)	27.3 (4.3)	28.2 (4.4)	.160	26.2 (4.8)	28.2 (4.7)	.001
Total Thigh Muscle Volume (L), mean (SD)	11.6 (1.7)	11.2 (1.6)	.063	7.4 (1.0)	7.6 (1.1)	.222
Physical Activity (min/week), mean (SD)	1173 (746)	881 (642)	.004	973 (560)	902 (589)	.322
SOMMA multimorbidity index, mean (SD)	0.62 (0.70)	0.94 (0.91)	.009	0.54 (0.73)	0.75 (0.84)	.040
SOMMA multimorbidity index, N (%)			.064			.104
0	41 (50%)	38 (36%)		79 (57%)	57 (47%)	
1	31 (38%)	43 (41%)		46 (33%)	44 (36%)	
2+	10 (12%)	25 (24%)		13 (9%)	21 (17%)	
Total medication use, mean (SD)	2.0 (2.2)	5.5 (3.5)	<.0001	2.8 (2.6)	5.6 (3.3)	<.0001
Total medication use, N (%)			<.0001			<.0001
0-2	55 (67%)	11 (10%)		73 (53%)	20 (16%)	
3-4	21 (26%)	37 (35%)		40 (29%)	37 (30%)	
5+	6 (7%)	58 (55%)		25 (18%)	65 (53%)	
Max OXPHOS (pmol/(s*mg)), mean (SD)	72.8 (22.6)	64.8 (19.7)	.011	58.3 (16.3)	58.9 (16.2)	.770
Max ETS (pmol/(s*mg)), mean (SD)	93.3 (26.6)	82.4 (22.1)	.003	76.7 (20.5)	76.1 (18.8)	.817
ATP Max (mM/sec), mean (SD)	0.62 (0.19)	0.54 (0.15)	.002	0.55 (0.16)	0.52 (0.11)	.029

Note. SOMMA = Study of Muscle, Mobility and Aging; OXPHOS = oxidative phosphorylation; ETS = electron transfer system; ATP = adenosine triphosphate

eTable 2: Skeletal muscle bioenergetic capacity by mito-modulatory medication use.

		Mito-Modulatory Medication Use					
		Men			Women		
Outcome	Model Number	No N=87	Yes N=110	p-value	No N=140	Yes N=124	p-value
**Max OXPHOS (pmol/(s*mg))**	M0	73.4 (67.7-79.0)	66.3 (61.2-71.4)	.0197	58.7 (55.5-61.8)	58.8 (55.6-62.1)	.9418
	M1	70.5 (63.7-77.3)	65.1 (58.8-71.4)	.0840	56.0 (52.2-59.8)	56.9 (53.0-60.9)	.6350
	M2	69.9 (63.1-76.7)	65.3 (59.0-71.6)	.1414	56.0 (52.2-59.8)	56.9 (52.9-60.8)	.6639
	M3	69.2 (62.4-76.1)	66.8 (60.3-73.4)	.4837	56.0 (52.1-59.8)	57.0 (52.9-61.1)	.6225
**Max ETS (pmol/(s*mg))**	M0	92.4 (85.8-99.0)	83.6 (77.6-89.5)	.0133	78.0 (74.0-82.0)	77.4 (73.2-81.5)	.7911
	M1	87.9 (80.0-95.7)	81.3 (74.0-88.6)	.0683	73.7 (69.1-78.4)	74.3 (69.4-79.1)	.8237
	M2	87.2 (79.3-95.1)	81.5 (74.2-88.8)	.1168	73.7 (69.1-78.4)	74.4 (69.5-79.3)	.7891
	M3	86.5 (78.6-94.4)	83.1 (75.5-90.7)	.3978	73.5 (68.8-78.3)	74.8 (69.7-79.8)	.6282
**ATP Max (mM/sec)**	M0	0.62 (0.58-0.65)	0.55 (0.51-0.58)	.0038	0.55 (0.53-0.58)	0.52 (0.49-0.54)	.0332
	M1	0.59 (0.54-0.63)	0.53 (0.49-0.58)	.0385	0.52 (0.49-0.55)	0.49 (0.46-0.52)	.1180
	M2	0.58 (0.54-0.63)	0.53 (0.49-0.58)	.0480	0.52 (0.49-0.55)	0.49 (0.46-0.52)	.1022
	M3	0.58 (0.53-0.62)	0.54 (0.50-0.59)	.2324	0.52 (0.49-0.55)	0.49 (0.46-0.52)	.1510

Note. OXPHOS = oxidative phosphorylation; ETS = electron transfer system; ATP = adenosine triphosphate.
M0: adjusted for technician (for Max OXPHOS and Max ETS) or site (ATP Max).
M1: adjusted for technician/site, age, race, BMI, physical activity.
M2: adjusted for technician/site, age, race, BMI, physical activity, SOMMA multimorbidity index.
M3: adjusted for technician/site, age, race, BMI, physical activity, SOMMA multimorbidity index, total medications.

eTable 3: ATP Max by mito-modulatory medication use.

Outcome	Model Number	Men			Women		
		No N=82	Yes N=106	p-value	No N=138	Yes N=112	p-value
**ATP Max (mM/sec)**	M0	0.62 (0.58-0.65)	0.55 (0.51-0.58)	.0038	0.55 (0.53-0.58)	0.52 (0.49-0.54)	.0332
	M1	0.59 (0.54-0.64)	0.53 (0.49-0.57)	.0162	0.52 (0.49-0.55)	0.49 (0.46-0.52)	.1213
	M2	0.59 (0.54-0.63)	0.53 (0.49-0.57)	.0197	0.52 (0.49-0.55)	0.48 (0.45-0.51)	.0311
	M3	0.59 (0.54-0.63)	0.53 (0.49-0.57)	.0297	0.52 (0.49-0.54)	0.49 (0.46-0.52)	.1266

Note. ATP = adenosine triphosphate.
M0: adjusted for site.
M1: adjusted for site, age, race, BMI.
M2: adjusted for site, age, race, muscle size.
M3: adjusted for site, age, race, BMI, muscle size.

eTable 4: Absolute standardized mean difference before and after inverse probability of treatment weighting analysis.

	Men				Women			
	**Before IPTW**	**p-value**	**After IPTW**	**p-value**	**Before IPTW**	**p-value**	**After IPTW**	**p-value**
Age (years)	0.22	0.039	0.14	0.411	0.00	0.851	0.00	0.995
White (%)	0.06	0.643	0.01	0.966	0.06	0.577	0.01	0.960
Body Mass Index (BMI)	0.21	0.160	0.21	0.123	0.42	0.001	0.02	0.881
Physical Activity (min/week)	0.42	0.004	0.13	0.355	0.12	0.322	0.02	0.859
SOMMA multimorbidity index	0.39	0.009	0.10	0.510	0.27	0.040	0.01	0.920
Total medication use	1.20	<.0001	0.03	0.881	0.94	<.0001	0.20	0.137

Note. ITPW = inverse probability of treatment weighting; SOMMA = Study of Muscle, Mobility and Aging

eTable 5: Skeletal muscle bioenergetic capacity by levels of mito-modulatory medication use.

		Mito-Modulatory Medication Use							
		Men				Women			
Outcome	Model Number	No medications N=87	1 medication N=54	2+ medications N=56	p-trend across the categories	No medications N=140	1 medication N=81	2+ medications N=43	p-trend across the categories
**Max OXPHOS (pmol/(s*mg))**	M0	73.5 (67.8-79.2)	67.7 (61.0-74.5)	65.1 (58.9-71.3)	.0209	58.7 (55.5-61.9)	58.8 (54.9-62.7)	58.9 (53.9-63.8)	.9439
	M1	70.6 (638-77.5)	65.9 (57.8-74.0)	64.6 (57.7-71.6)	.1048	56.0 (52.2-59.8)	56.8 (52.3-61.3)	57.2 (51.8-62.5)	.6724
	M2	69.7 (62.7-76.6)	64.3 (56.1-72.6)	65.9 (58.8-73.0)	.3415	56.0 (52.2-59.8)	56.8 (52.2-61.3)	57.1 (51.7-62.5)	.7036
	M3	68.7 (61.7-75.8)	65.2 (56.9-73.5)	68.0 (60.4-75.5)	.8623	55.9 (52.1-59.8)	56.9 (52.3-61.5)	57.3 (51.7-62.8)	.6598
**Max ETS (pmol/(s*mg))**	M0	92.6 (86.0-99.2)	86.2 (78.3-94.1)	81.5 (74.3-88.7)	.0088	78.1 (74.0-82.1)	78.2 (73.3-83.2)	75.9 (69.6-82.1)	.5274
	M1	88.2 (80.3-96.2)	83.2 (73.8-92.5)	80.1 (72.1-88.2)	.0602	74.3 (69.5-79.0)	75.3 (69.6-80.9)	73.2 (66.4-79.9)	.7506
	M2	87.2 (79.2-95.3)	81.5 (72.0-89.7)	81.5 (73.2-89.7)	.2100	74.3 (69.5-79.0)	75.3 (69.6-81.0)	73.2 (69.5-79.0)	.7677
	M3	86.3 (78.1-94.4)	82.4 (72.8-92.1)	83.6 (74.8-92.4)	.5935	73.8 (69.0-78.7)	75.9 (70.2-81.7)	74.2 (67.2-81.1)	.9350
**ATP Max (mM/sec)**	M0	0.62 (0.58-0.65)	0.56 (0.52-0.61)	0.53 (0.48-0.57)	.0029	0.55 (0.53-0.58)	0.51 (0.48-0.54)	0.52 (0.48-0.57)	.2328
	M1	0.59 (0.55-0.64)	0.55 (0.50-0.61)	0.52 (0.47-0.57)	.0156	0.52 (0.49-0.55)	0.49 (0.45-0.52)	0.50 (0.46-0.55)	.5786
	M2	0.59 (0.54-0.63)	0.54 (0.48-0.60)	0.53 (0.48-0.58)	.0773	0.52 (0.49-0.55)	0.49 (0.45-0.52)	0.50 (0.46-0.55)	.5244
	M3	0.58 (0.53-0.63)	0.54 (0.49-0.60)	0.54 (0.49-0.60)	.3299	0.52 (0.49-0.55)	0.49 (0.45-0.52)	0.50 (0.46-0.55)	.6013

Note. OXPHOS = oxidative phosphorylation; ETS = electron transfer system; ATP = adenosine triphosphate.
M0: adjusted for technician (for Max OXPHOS and Max ETS) or site (ATP Max).
M1: adjusted for technician/site, age, race, BMI, physical activity.
M2: adjusted for technician/site, age, race, BMI, physical activity, SOMMA multimorbidity index.
M3: adjusted for technician/site, age, race, BMI, physical activity, SOMMA multimorbidity index, total medications.
